# Prevalence and factors associated with adequate dietary diversity among pregnant women in Nekemte town, Western Ethiopia, 2021

**DOI:** 10.3389/fnut.2023.1248974

**Published:** 2023-12-15

**Authors:** Haile Bikila, Berhanu Tessisa Ariti, Meseret Belete Fite, Jabessa Hatahu Sanbata

**Affiliations:** ^1^Department of Public Health, Institute of Health Sciences, Wollega University, Nekemte, Ethiopia; ^2^Nekemte Health Science College, Nekemte, Ethiopia

**Keywords:** dietary diversity, pregnant women, factors associated, dietary adequacy, Nekemte Twon

## Abstract

**Background:**

Dietary diversity refers to increasing the consumption of a variety of foods. The consumption of diversified food during pregnancy enables the adequate intake of 11 important micronutrients. Inadequate dietary intake during pregnancy is the major determinant factor in the risk of low birth weight infants. It is capable of ensuring the adequate intake of essential nutrients, which can promote good physical health and mental development. Pregnant women require more protein, iron, iodine, vitamin A, folate, and other nutrients. Adequate intake of fruit, vegetables, and animal products throughout the life cycle helps ensure that women enter pregnancy and lactation without deficiencies. Micronutrient deficiency and protein, carbohydrate, and fat intake imbalances are also linked to an increased risk of chronic disease.

**Objective:**

To assess the prevalence and associated factors with adequate dietary diversity among pregnant woman in Nekemte town, western Ethiopia, 202.1.

**Methods:**

A community-based cross-sectional study was conducted among 475 pregnant women in the town. We used a systematic random sampling technique. Data were collected through face-to-face interviews by trained data collectors using a validated questionnaire. Before being exported to STATA version 14, data were entered into EpiData version 3.1, cleaned, coded, and checked for missing values. Results from bivariable analysis of *p*-value less than 0.25 were moved to a multivariable binary logistic regression model for analysis. Finally, multivariable logistic regression with *p*-value of less than 0.05 was considered statistically significant.

**Results:**

The Prevalence of adequate dietary diversity was 43.6% (95% CI; 39.1–48.1). Households with the richest wealth index adjusted odds ratio (AOR = 3.17; 95%Confidence Interval = 1.60–6.28), those who have antenatal care (AOR = 2.16; 95%CI = 1.22–3.84), and women who were government employees (AOR = 1.87; 95%CI = 1.01–3.48) were positively associated with adequate dietary diversity. On the other hand, food-insecure households (AOR = 0.34; 95%CI = 0.17–0.66), women who had not changed their meal frequency (AOR = 0.613; 95%CI = 0.38–0.99), and women in their third trimester (AOR = 0.40; 95%CI = 0.20–0.81) were negatively associated with adequate dietary diversity during pregnancy.

**Conclusion:**

The findings showed that there was a low acceptable level of dietary diversity among pregnant women in the town. Wealth index, antenatal care, women’s occupation, household food insecurity, gestational age, and not changing meal frequency were identified as factors associated with adequate dietary diversity. Therefore, multi-sectoral collaboration is needed to enhance the dietary diversity of pregnant women by promoting women’s employment and strengthening sustainable income-generating activities.

## Introduction

Diet refers to how people feed themselves and the foods they consume, which are heavily influenced by people’s traditions, religion, economic position, social status, and the opportunities provided by their natal surroundings (1). Dietary diversity refers to increasing the consumption of a variety of foods (2). It is capable of ensuring the adequate intake of essential nutrients, which can promote good physical health and mental development (3, 4). Pregnant women require more protein, iron, iodine, vitamin A, folate, and other nutrients (5, 6). The adequate intake of fruit, vegetables, and animal products throughout the life cycle helps to ensure that women enter pregnancy and lactation without deficiencies (6). Micronutrient deficiency and protein, carbohydrate, and fat intake imbalances are also linked to an increased risk of chronic disease (7). In resource-limited settings across the globe, low-quality, monotonous diets are the norm. When grain or tuber-based staple foods dominate and diets lack vegetables, fruits, and animal-source foods, the risk of a range of micronutrient deficiencies is high (8, 9). Worldwide, maternal undernutrition is the leading developmental challenge affecting nearly half of the world’s population and is responsible for the deaths of 3.5 million mothers and children every year. It accounts for approximately 7% of the disease burden and contributes to an increased chance of poor pregnancy outcomes (10). Worldwide, inadequate dietary diversity is a major public health issue for pregnant women (11). Deficiencies in key macronutrients and micronutrients have a substantial impact on pregnancy outcomes and neonatal health (12). Deficiencies of certain nutrients are also associated with fetal and newborn deaths, birth defects, and decreased physical and mental potential in the child (5, 6). Evidence suggests that the effects of fetal nutrition may persist well into adulthood, with possible intergenerational effects (13). A large study conducted in four countries reported that only 20% of pregnant women in Pakistan, a quarter (25%) of pregnant women in the Democratic Republic of the Congo (DRC), half (50%) of pregnant women in Guatemala, and 70% of pregnant women in India have adequate dietary diversity (14). In sub-Saharan African countries, diets are predominantly based on starchy foods with little or no animal products and few fresh fruits and vegetables (15). In Tanzania, cereals and starchy roots contribute 51 and 19% of the total dietary energy supply and 71 and 61% of the population’s dietary use of maize and rice, respectively (16). The least consumed foods in Tanzania include eggs, fish, flesh meat, organ meat, milk and milk products, other fruits, and vitamin A-rich fruits (16). Associated factors that were identified for suboptimal dietary practice were women’s age, education, occupation, use of medical services, socioeconomic status, previous delivery, morbidity, household assets, land ownership, and daily meal frequency (17).

A national-level study in Ethiopia (18) discovered a low rate of adequate dietary diversity (41%) among pregnant women. A large study that involved 540 pregnant women revealed that the mean dietary diversity score was 2.85 (19). Evidence from studies conducted in Ethiopia documented that the low Prevalence of adequate dietary diversity among pregnant women ranged from 25.4% in Shashemane town to 65.5% in the Illu Aba Bor zone (20–24). Studies have reported that the dietary diversity of pregnant women is associated with many factors. Among these, maternal education, nutritional information, dietary diversity knowledge, household wealth index level, and not having additional meals during pregnancy were factors significantly associated with pregnant women’s dietary diversity practice in Ethiopia (25 and 26).

Even though there have been reports about the dietary diversity of pregnant women, most of the existing reports are institutional-based. Because of this, identifying community-based specific factors related to dietary diversity in pregnant women is critical to designing evidence-based, appropriate long-term nutritional intervention strategies that would optimize pregnancy and fetal outcomes. This was in line with the current emphasis on the first 1,000 days of life as a window of opportunity to promote healthy child growth by updating data, which is essential for developing effective intervention strategies (20).

A limited study with a community-based design was previously conducted to assess the prevalence and factors associated with adequate dietary diversity among pregnant women in the study area, as well as how food varies according to culture, religion, and food anthropology, which needs further investigation. The study may be useful for the Nekemte Town Health Office in prioritizing, designing, and initiating intervention programs. Therefore, the aim of this study was to assess the prevalence and factors associated with adequate dietary diversity among pregnant women in Nekemte town.

## Methods and materials

### Study setting

Nekemte is located 330 km west of Addis Ababa, the capital of Ethiopia. Based on the population projection, the town has a total population of 135,856, of which, 4,714 (3.47%) are pregnant women. Nekemte is the capital city of the East Wollega zone of Oromia Regional State. The town has two government health centers, two referral hospitals, and many private health facilities. The town’s altitude ranges from 1960 to 2,170 meters above sea level; its average annual rainfall is 1854.9 mm and the average temperature ranges from 14°C to 26°C. Agriculture is the dominant livelihood of the population in the study area. The major crops grown in the area of Nekemte include cereals, pulses, and horticultural crops such as fruit, vegetables, and root crops. Cereals include maize, sorghum, teff, wheat, and barley, and pulses include beans, peas, field peas, lentils, and vetch. Furthermore, oilseeds such as linseed and *Guizotia abyssinica* are growing in small quantities (21).

### Study design

Community-based cross-sectional.

### Study Period

From October 15–25, 2021.

### Population

The source population was comprised of pregnant women who resided in Nekemte town during the study period.

### Study population

The study population was comprised of pregnant women who lived in randomly selected sub-cities.

### Inclusion and exclusion criteria

**Inclusion criteria**: Pregnant women who were in Nekemte town during the study period.

**Exclusion criteria**: Pregnant women who were unable to communicate due to mental status or serious illness.

**Sample Size Determination**: For the first specific objective, the sample size was computed using a single population proportion formula. From a previously conducted study in Shashemane, 25.4% of the study participants had a high dietary diversity score with a 5% margin of error and a 95% confidence level (22).


$$\frac{N=\;{\left(Z_{1-A/2}\right)}^{2}Pq}{D^{2}}$$

N = required sample size.

P = Probability of having a highly diversified diet (25.4%).

Q = Probability of not having a diversified diet (1–25.4%).

D = Margin of Error = 5% = (0.05).


$$\frac{N=\;{\left(1.96\right)}^{2}0.254^{*}0.746=292}{\;0.05^{2}}$$

For the second specific objective of the study, Epi-Info software statistics were employed for sample size calculation, using 80% power and a 95% confidence interval, according to studies conducted in the Ilu Ababori zone, Shashemene town, and west Gojjam zone. Since the sample size of the first objective is greater than that of the second objective, a sample size of 292 was implemented for pregnant mothers in this study. Using a design effect of 1.5 and a response rate of 10%, the total sample size for this study was 482.

### Sampling procedure

Three sub-clusters were selected using a simple random sampling method among seven sub-clusters in Nekemte town. From this sub-cluster, 482 pregnant women were proportionally allocated to the three selected sub-cities. We identified all eligible pregnant women through house-to-house visits with the help of health extension workers. The total number of households with pregnant women was accessed in selected sub-cities of the town. After the sampling interval (K) was calculated, households with eligible pregnant women were selected using a systematic random sampling technique. We selected pregnant women using the lottery method for households with more than one pregnant woman. If the pregnant women were not at home during the first visit, the data collators visited their house the next day; the pregnant women who were not available during the second visit were recorded as non-responsive. First, the initial pregnant woman was selected from the eligible group randomly, and then subsequent pregnant women were selected systemically. Then, pregnant women were selected at every Kth value until the sample size was fulfilled. All selected pregnant women were interviewed through house-to-house visits by data collectors.

### Data collection tools and techniques

Prior to data collection, experienced and skilled data collectors were selected. The tools used in the study were adapted to different literature with similar objectives and demographic health surveys (DHS). Before the actual data collection, a questionnaire to collect demographic, health, and dietary data was prepared in English and then translated into Afan Oromo. After some corrections to the original tools had been made, the final version of the questionnaire was again translated into Afan Oromo.

Four nurses with Bachelor of science (BSc) degrees in nursing were involved in data collection. Two BSc health professionals were recruited for supervision. Data completion and accuracy were checked each day during data collection. Before interviews, informed voluntary written and signed consent was obtained. Initially, participants were asked to complete a brief questionnaire related to demographics, obstetrics, health care, food taboos, home food insecurity, food habits, and nutritional knowledge. Household food security status was assessed using the Household Food Insecurity Access Scale (HFIAS) (23). Household food security was categorized as food insecure for those who scored 2 or above out of 27 household food insecurity indicators and as food secure for pregnant women who scored less than 2 out of 27 household food insecurity indicators (23). Data were collected using interviewer-administered questionnaires and the 24-h recall method adapted from various literature, primarily Food and Agriculture Organization (FAO) guidelines for measuring household and individual dietary diversity (24). A total of 10 food groups were considered in this study, i.e., cereals (grains, white roots and tubers, and plantain), pulses (beans, peas, and lentils), nuts and seeds, dairy products, meat (any animal meat products, poultry, and fish), eggs, dark green leafy vegetables, other vitamin A-rich fruits and vegetables, other vegetables, and other fruits. The principal investigator managed the overall supervision and coordination (21, 25).

A wealth index was employed to estimate the economic level of the families. The wealth dispersion was generated by applying principal component analysis. The index was calculated using 17 household variables, including ownership of a latrine, selected household assets, housing standard, and source of water used for drinking and cooking. Lastly, the wealth data was categorized into quintiles: poorest, poor, medium, rich, and richest (26).

### Data quality assurance

The data collectors were by the principal investigator. The training emphasized the data collection tool, how to handle the data, and the privacy and confidentiality of the information collected.

A pre-test was conducted on 10% of the total sample in the Burka Jato sub-city, the nonselected setting, to ensure data quality and test the competency of the data collectors. Data collection was also conducted under close supervision. Proper categorization and coding of the data was maintained for quality and all data were checked for completeness, accuracy, and clarity by the principal investigator and supervisors immediately after the data were collected.

### Data processing and analysis

Data were entered into EpiData version 3.1, cleaned, coded, and checked for missing data and outliers before being exported to STATA version 14 for further analysis. Descriptive statistics were done to summarize the data. Frequencies, cross-tabulation, and percentages were calculated for all the categorical variables. The goodness of fit was checked by the Hosmer-Lemeshow statistic and omnibus tests. All the independent variables were entered into the equation first, and each one was removed one at a time if they did not contribute to the regression equation. All variables with *p* < 0.25 in the bivariable analyses were included in the final model of multivariable analysis to control all possible confounders. A multico-linearity test was carried out to show the correlation between independent variables using the collinearity statistics (variance inflation factors >10 were considered suggestive of the existence of multico-linearity). Backward elimination was used. Explanatory variables significantly associated with outcome variables with a value of *p* < 0.05 in multivariable analysis were determined as factors associated with adequate dietary diversity. To measure the strength of the association between predictors and outcome variables, odds ratios with a 95% confidence interval were calculated.

### Dependent variable

Adequate dietary diversity status.

### Independent variables

**Socio-demographic factors**: age, marital status, occupation, maternal education, religion, ethnicity, family size, wealth index.

**Household food security status**: food-secure household or food - insecure household.

**Pregnancy and healthcare-related factors**: early marriage, birth interval, parity, illness during pregnancy, history of complications, antenatal care (ANC) visit, history of multiple pregnancies, gestational age.

**Food habits**: meal frequency/size, food taboos, food cravings, food aversions, pica, substance use (smoking and alcoholism), coffee consumption, nutritional knowledge.

### Operational definition and definition of terms

**Dietary Diversity Status**: Number of food groups consumed by pregnant women out of the 10 food groups; food groups include starch staples, pulses, nuts, any meat products (including poultry and fish), dark green leafy vegetables, vitamin A-rich vegetables and fruits, and other vegetables and fruits (21).

**Adequate Dietary Diversity**: Dietary diversity score of pregnant women receiving at least 5 food groups out of 10 (21, 25).

**Inadequate Dietary Diversity**: When pregnant women consumed less than 5 of the 10 food groups within 24 h before the survey at third trimester (21, 25).

**Good Dietary Knowledge**: Women had good dietary knowledge if they scored greater than or equal to the mean.

**Poor Dietary Knowledge**: Women had poor dietary knowledge if they scored less than the mean (21, 25).

**Food-Insecure Household**: Pregnant women who scored 2 and above out of 27 household food insecurity access scale indicator (23).

**Food-Secure Household**: Pregnant women who scored less than 2 out of 27 household food insecurity indicators (23).

### Ethical considerations

Prior to data collection, ethical approval was obtained from Wollega University Institute of Health Sciences, postgraduate study, ethical review committee. An official letter was written to the Nekemte town health department. After permission was obtained from the town health department, a permission letter would be given to the sub-city administrative office. Health facility management and staff were also informed about the purpose of the research. Finally, written consent was obtained from individual study participants. The purpose and objective of the research were clearly explained to the study participants. In addition, the study participants were assured of confidentiality. They were also assured that in the event they want to withdraw from the interview at any point, they would be free to do so and there would be no penalty for this action.

## Results

### Socio-demographic characteristics

A total of 482 pregnant women were eligible and 475 consented, giving a response rate of 98.5%. The mean age of the women was 27.9 (±5.2), ranging from 16 to 42. The majority of respondents, 438 (92.2%), were married, 266 (56.0%) of them had received a formal education (Grade 1–12), and half of them, 263 (55.7%), were Protestant (Table 1).

**Table 1:** Socio-demographic characteristics of pregnant women in Nekemte town, Western Ethiopia, October 2021 (*n* = 475).

Variables	Frequency (*n*)	Percentage (%)
Age (years)		
<24	105	22.1
24–35	310	65.3
>35	60	12.6
Mean (± SD)	27.9 (± 5.2)	
Maternal marital status		
Single	8	1.7
Divorced	17	3.6
Married	438	92.2
Widowed	12	2.5
Maternal educational status		
No formal education	26	5.5
Elementary school (1–8)	74	15.9
High School (9–12)	192	40.4
College and above	183	38.5
Maternal occupation		
Housewife	200	42.1
Merchant	98	20.6
Daily Laborer	38	8.0
Governmental employees	111	23.4
Student	28	5.89
Ethnicity		
Oromo	402	84.6
Amara	49	10.3
Gurage	24	5.1
Age at marriage (years)		
<18	21	4.4
18–24	369	77.7
>24	85	17.9
Time to reach nearest health facility		
<30 min	414	87.2
≥30 min	61	12.8
Wealth index		
Poorest	109	23.0
Poor	82	17.3
Middle	95	20.0
Rich	95	20.0
Richest	94	19.8
Family size		
≤ 3	170	35.8
4–5	207	43.58
>5	98	20.6
Household food security status		
Secure	59	12.4
Insecure	416	87.6

### Pregnancy and feeding pattern related characteristics

Approximately 221 (46.53%) pregnant women were in the second trimester of pregnancy during the assessment. More than three-forth, 374 (78.74%), of pregnant women reported that they had attended an antenatal care visit. Approximately 286 (60.2%) pregnant women consumed four or more meals per day on the last day before assessment. On the other hand, 66 (13.89%) pregnant women had had complications during their current pregnancy (Table 2).

**Table 2:** Pregnancy and feeding pattern-related characteristics of pregnant women Nekemte town, Western Ethiopia, October 2021 (*n* = 475).

Characteristics	Category	Frequency	Percent
Number of pregnancy	≤2	302	63.6
	3–4	160	33.7
	≥5	13	2.7
Parity	0	184	38.7
	1–2	239	50.3
	≥3	52	10.9
Birth interval (months)	12–36	228	48.0
	>36	247	52.0
Gestational Age	First trimester (< 16 weeks)	77	16.2
	2nd trimester (16–27 weeks)	221	46.5
	3rd trimester (≥28 weeks)	177	37.5
Age at first pregnancy	<24 years	238	50.1
	≥24 Years	237	49.9
ANC	Had not attended	101	21.3
	Had Attended	374	78.7
Complications during current pregnancy	No	409	86.1
	Yes	66	13.9
Food taboos	No	416	87.9
	Yes	59	12.4
Craving	No	286	60.2
	Yes	189	39.8
Food aversion	No	368	77.5
	Yes	107	22.5
Food Pica	No	437	92.0
	Yes	38	8.0
Fasting	No	433	91.2
	Yes	42	8.8
Meal frequency	<4	189	39.8
	≥4	286	60.2
Maternal nutritional knowledge	Poor	285	60.0
	Good	190	40.0

### Prevalence of adequate dietary diversity

**Figure 1:**
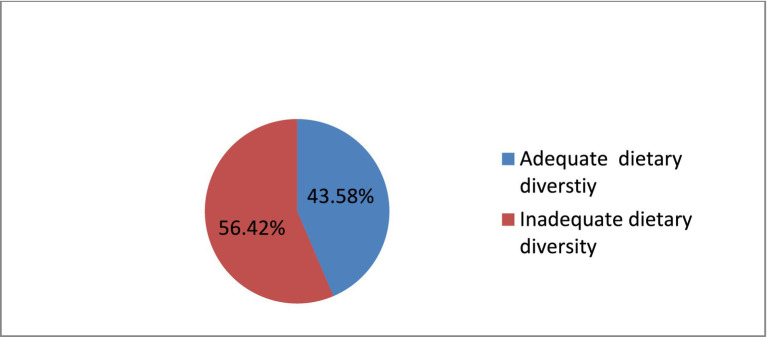
Prevalence of adequate dietary diversity among pregnant women in Nekemte town, Western Ethiopia, October 2021 (*n* = 475).

The mean dietary diversity score was 4.29 ± 1.18. Of the total respondents, 43.58% (95% CI = 39.10–48.05) had adequate dietary diversity (Figure 1).

### Food consumption patterns

**Figure 2:**
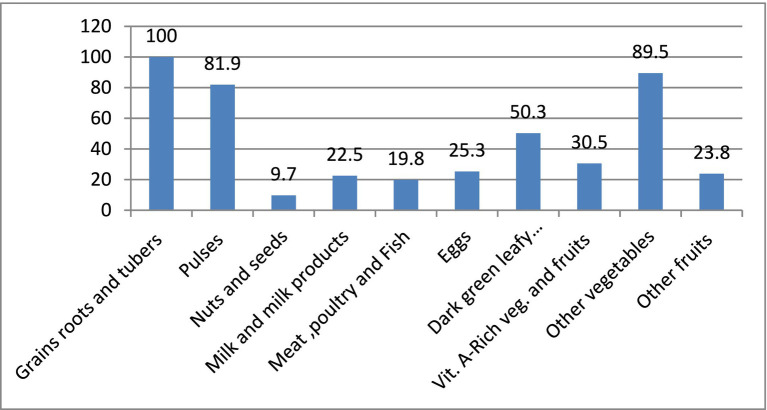
Food consumption patterns of pregnant women in Nekemte town, Western Ethiopia, October 2021 (*n* = 475).

In terms of food groups consumed by pregnant women in the previous 24 h, 100% consumed starchy stable food groups, 89.5% consumed other vegetable food groups, 81.9% consumed pulse food groups, 50.3% consumed dark green leafy vegetables, and 30.5% consumed other vitamin-A-rich fruits and vegetables, while the least-consumed food groups were fish, poultry, and meat (19.8%) and nuts and seeds (9.7%) (Figure 2).

### Factors associated with adequate dietary diversity

In the bivariable analysis, maternal nutritional knowledge, wealth index, household food insecurity, maternal age, maternal educational status, maternal occupation, family size, gestational age, antenatal visits, food taboos, gravida, and food frequency were found to be candidates for multivariable analysis at value of *p* < 0.25. The Hosmer and Lemeshow goodness of fit gave *p* = 0.734, suggesting evidence of the goodness of fit of the model.

Adequate dietary diversity was 3.2 times (AOR = 3.17; 95%CI = 1.60–6.28) more likely among the rich quintiles compared to the poorest, 2.2 times (AOR = 2.16; 95%CI =1.22–3.84) more likely among those who had attended antenatal care compared to their counterparts, and almost 2 times (AOR = 1.87; 95%CI =1.01–3.49) more likely among government employees compared to housewives. However, adequate dietary diversity in pregnant women was found to be 66.2% (AOR = 0.34; 95% CI = 0.17–0.66) less likely in food-insecure households, 60% (AOR = 0.40; 95% CI = 0.20–0.81) less likely in third-trimester pregnancy compared to first-trimester pregnancy, and 38.7% (AOR = 0.61; 95% CI = 0.38–0.99) less likely in women who had not changed their meal frequency compared to those who had increased meal frequency (Table 3).

**Table 3:** Multivariable analysis of factors associated with adequate dietary diversity in pregnant women in Nekemte town, Western Ethiopia, October 2021 (*n* = 475).

Variables	Dietary diversity		COR (95%CI)	AOR (95%CI)	value of *p*
	Adequate (207)	Inadequate (268)			
Maternal nutritional knowledge					
Poor	109 (52.7)	176 (65.7)	1	1	
Good	98 (34.3)	92 (47.3)	1.720 (1.19–2.49)	1.304 (0.85–1.99)	0.27
Wealth Index					
Poorest	26 (12.6)	83 (31.0)	1	1	
Poor	35 (16.9)	47 (17.5)	2.378 (1.28–4.42)	3.10 (1.55–6.20)	0.001
Middle	41 (19.8)	54 (20.2)	2.42 (1.33–4.41)	2.20 (1.13–4.28)	0.02
Rich	54 (26.1)	41 (15.3)	2.20 (2.31–7.65)	3.17 (1.60–6.28)	<0.001**
Richest	51 (24.6)	43 (16.0)	3.79 (2.08–6.89)	2.54 (1.26–5.10)	0.01
Maternal household food security					
Secure	18 (19.8)	41 (6.7)	1	1	
Insecure	166 (80.2)	250 (93.2)	0.292 (0.16–0.53)	0.34 (0.17–0.66)	<0.001**
Maternal age (years)					
<24	40 (19.3)	65 (24.3)	1	1	
24–35	142 (68.6)	168 (62.7)	0.82 (0.49–1.38)	1.01 (0.57–1.79)	0.98
>35	25 (12.1)	35 (13.1)	0.69 (0.40–1.18)	0.69 (0.29–1.64)	0.40
Maternal educational status					
No formal Educ.	8 (3.9)	18 (6.7)	1	1	
Elementary school (1-8)	23 (11.1)	51 (19.03)	1.02 (0.39–2.67)	1.12 (0.39–3.27)	0.83
High School (9-12)	82 (39.6)	110 (41.0)	1.68 (0.70–4.05)	1.26 (0.48–3.33)	0.64
College & above	94 (45.4)	89 (33.21)	2.38 (0.98–5.74)	1.14 (0.45–3.46)	0.681
Maternal occupation					
Housewife	69 (33.3)	131 (48.9)	1	1	
Merchant	47 (22.71)	51 (19.03)	1.75 (1.07–2.86)	1.55 (0.87–2.64)	0.15
Daily Laborer	15 (7.3)	23 (8.6)	1.24 (0.61–2.53)	2.07 (0 0.93–4.63)	0.08
Student	11 (5.3)	17 (6.3)	1.23 (0.55–2.77)	1.13 (0.45–2.81)	0.80
Government employee	65 (30.9)	46 (16.8)	2.68 (1.67–4.32)	1.87 (1.01–3.48)	0.047*
Maternal family size					
≤ 3	61 (29.5)	109 (40.5)	1	1	
4–5	103 (49.5)	(104 38.8)	1.77 (1.17–2.68)	1.59 (0.93–2.71)	0.09
>5	43 (20.8)	55 (20.5)	1.40 (0.84–2.32)	0.95 (0.49–1.86)	0.10
Gestational age					
First trimester (< 16 weeks)	38 (18.4)	39 (14.6)	1	1	
Second trimester (16–27 weeks)	98 (47.3)	123 (45.9)	0.82 (0.49–1.38)	0.56 (0.29–1.05)	0.073
Third trimester (≥28 weeks)	71 (34.3)	106 (39.6)	0.69 (0.40–1.18)	0.40 (0.20–0.81)	0.01*
Antenatal care					
Had not attended	33 (15.9)	68 (25.4)	1	1	
Had Attended	174 (84.1)	200 (74.6)	1.792 (1.13–2.85)	2.16 (1.22–3.84)	0.01*
Food taboos					
No	177 (85.51)	239 (89.2)	1	1	
Yes	30 (14.5)	29 (10.8)	1.40 (0.81–2.41)	1.542 (0.85–2.81)	0.16
Gravida					
≤2	125 (60.4)	177 (66.0)	1	1	
3–4	78 (37.7)	82 (30.6)	1.35 (0.92–1.98)	1.087 (0.63–1.87)	0.76
≥5	4 (1.9)	9 (3.4)	0.63 (0.19–2.09)	0.915 (0.214–3.91)	0.91
Food frequency					
Increased	121 (58.5)	123 (45.9)	1	1	
No change	62 (29.9)	110 (41.1)	0.57 (0.38–0.86	0.61 (0.38–0.99)	0.044
Decreased	24 (29.9)	35 (41.1)	0.70 (0.39–1.24)	0.77 (0.39–1.49)	0.43

COR, crude odd ratio; AOR, adjusted odd ratio.

**Statistically significant at value of *p* < 0.001.

*Statistically significant at value of *p* < 0.05.

## Discussion

The aim of this study was to assess the prevalence and factors associated with adequate dietary diversity among pregnant women in Nekemte Town. The study investigated the dietary diversity status of pregnant women in Nekemte town, which showed that the prevalence of adequate dietary diversity was 43.6% (95% CI: 39.1–48.1%). Moreover, wealth index, antenatal care, occupation, household food insecurity, gestational age, and meal frequency were identified as factors associated with adequate dietary diversity among pregnant women in Nekemte town.

The study indicated that the overall status of adequate dietary diversity was found to be low. This study’s findings are nearly identical to previous studies reported in Nepal (45%) (27); Wachamo town, Southern Ethiopia (42.6%) (28); and Dire Dawa city administration, Ethiopia (43%) (29). The results of this study revealed that the rate of adequate dietary diversity is lower than the studies carried out in Pakistan (89%); Ghana (79.9%); Illu Aba Bor zone, Southwest Ethiopia (65.5%); and Jimma zone south-west Ethiopia (56.4%) (27, 30–32). However, the prevalence of the present study are considerably higher than the reported rates in Gojjam north Ethiopia (19.9%) and northeast Ethiopia (31.4%) (33, 34). This discrepancy might be because of socio-demographic, socioeconomic, and seasonal variations.

The current study revealed that household wealth was significantly associated with adequate dietary diversity among pregnant women. This finding is in agreement with studies carried out in Ethiopia (30), Bangladesh (35), and Ghana (31), whereby adequate dietary diversity was reportedly more likely among the rich compared to the poorest quintiles. Also, a previous study done in Ethiopia showed that, there are increased odds of dietary diversity practiced by 85% in rich pregnant women than in poor pregnant women (33). Therefore, households with the richest wealth index have a better chance of having diversified diets; a possible reason for this is that higher income is associated with increased purchasing power, which can help promote dietary diversity.

Another factor that was found to be associated with adequate dietary diversity among pregnant women was household food insecurity.

Mothers from food-insecure households were found to be less likely to consume a diversified diet compared to their counterparts. This result is similar to the findings of studies conducted in Ethiopia (28, 30, 33, and) and Ghana (31), which revealed women from households experiencing food insecurity were less likely to achieve adequate dietary diversity. This might be related to the low availability and accessibility of adequate food in food-insecure households. Another possible reason may be that mothers in food-insecure households are likely to reduce their food intake to provide for their infants and small children, which leads to the mother’s inadequate dietary diversity.

In this study, pregnant women who had attended antenatal care were more likely to consume diversified food groups compared with their counterparts. Other studies carried out in Ethiopia showed that pregnant women who had ANC visit were more likely to attain adequate dietary diversity than those who had not (36, 37). Furthermore, a study conducted in Ghana investigated that frequent ANC attendance was a significant independent predictor of maternal dietary diversity (31). This might be explained by the fact that when pregnant women attend ANC, they receive nutrition information from health professionals and follow healthy dietary practices.

The findings of the present study are in agreement with several studies in Ethiopia that reported that women who had not yet started to consume an additional meal in pregnancy were less likely to have an adequate diversified diet compared to those who had started consuming additional meals (23, 27). Another study in the east Gojjam zone of northwest Ethiopia and Hossana town, south Ethiopia found that increasing meal frequency improves women’s dietary diversity (36, 37). This might be because pregnant women who consume an additional meal per day have a greater chance of accessing different food groups.

Similar to reports from Raya Azebo zone, Tigray region, Ethiopia (38), Kenya (17), and Nepal (27), the occupational status of pregnant women was found to be significantly associated with adequate dietary diversity in this study setup. This study showed that pregnant women who were government employees had higher dietary diversity compared to those who were housewives. Housewives are more confined to household work and are more financially dependent on their families and partners than government employees. This might be due to women who participate in yielding their family financial income having a better chance of earning income and better access to diversified foods and appropriate diets. Being in the third trimester was negatively associated with adequate dietary diversity. This relationship was also reported by another study carried out in Dessie town, northeastern Ethiopia (34).

Many women report that they eat better at the start of pregnancy (what they eat becomes more important); however, they do not always maintain the changes throughout pregnancy for unknown reasons (39). The possible reason might be that mothers adhere to specific diets because they habituate and adapt to the specific diets they prefer.

## Conclusion

This study has revealed a low prevalence of adequate dietary diversity among pregnant women. Adequate dietary diversity was positively associated with households with the richest wealth index, women who had attended antenatal care, and pregnant women who were government employees. However, adequate dietary diversity was negatively associated with food-insecure households, late gestational age (third trimester), and no changes in meal frequency during pregnancy.

## Recommendations

Nutritional education intervention needs to be tailored to meet the needs of pregnant women to improve their food intake frequency and the variety in their diet. Appropriate dietary intake and meal frequency counseling during ANC, delivery, and postnatal care are very important for improving dietary diversity during pregnancy. Support is needed for strengthening saving habits and establishing small-scale enterprises to create off-arm income opportunities to improve pregnant women’s buying power, leading to adequate dietary diversity. All development sectors need to collaborate to improve the dietary diversity and nutritional status of pregnant women through promoting and strengthening sustainable income-generating activities and saving strategies to improve the wealth status of pregnant women.

## Data availability statement

The raw data supporting the conclusions of this article will be made available by the authors, without undue reservation.

## Ethics statement

The studies involving human participants were reviewed and approved by the Wollega University Ethical Review Board. The patients/participants provided their written informed consent to participate in this study.

## Author contributions

HB, BA, MF, and JS were involved in the study conception, study design, statistical analysis, and results interpretation. All authors contributed to the article and approved the submitted version.

## Glossary

ANC: Antenatal care
AOR: Adjusted Odd Ratio
CI: Confidence Interval
COR: Crude Odd Ratio
DDS: Dietary Diversity Score
EAR: Estimated Average Requirement
FANTA: Food and Nutrition Technical Assistance
FAO: Food and Agriculture Organization of the United Nations
FHI: Family Health International
LMIC: Lower-Middle Income Countries
MDD: Minimum Dietary Diversity
MDD-W: Minimum Dietary Diversity for Women of Reproductive Age
POU: Prevalence of Undernourishment
STATA: Statistics Data
UNICEF: United Nations International Children’s Fund
USAID: United State Aid For International Development
WRA: Women of Reproductive Age
